# Partner Care Arrangements and Well-Being in Mid- and Later Life: The Role of Gender Across Care Contexts

**DOI:** 10.1093/geronb/gbab209

**Published:** 2021-11-09

**Authors:** Ginevra Floridi, Nekehia T Quashie, Karen Glaser, Martina Brandt

**Affiliations:** 1 Nuffield College, University of Oxford, Oxford, UK; 2 Department of Global Health & Social Medicine, King’s College London, London, UK; 3 Faculty of Social Sciences, TU Dortmund University, Dortmund, Germany

**Keywords:** Caregiver stress, Formal care, Informal care, Long-term care, SHARE

## Abstract

**Objectives:**

We assess gender moderation in the association between partner care arrangements and individuals’ well-being, and the extent to which gender differences vary across European care contexts.

**Methods:**

We use 2015 data from the Survey of Health, Ageing and Retirement in Europe for 3,465 couples aged 50+, where at least 1 partner receives care. We assess gender differences in individuals’ life satisfaction and depressive symptoms across 5 partner care arrangements: solo-; shared formal; shared informal; outsourced formal; and outsourced informal care. We explore heterogeneity in the gendered associations across 4 care contexts: Northern, Western, Southern, and Eastern Europe.

**Results:**

Sharing care with formal providers is associated with lower well-being among women than men, with a significant well-being “penalty” among Southern European women with partners in shared formal care. Outsourcing partner care to informal providers is associated with higher well-being than other care arrangements for men across care contexts, but with lower well-being for women in Southern Europe.

**Discussion:**

Policies to support caregivers’ well-being need to be sensitive to the coordination of formal and informal caregiving support for men and women in their respective care contexts.

Across aging societies, increasing proportions of individuals live with partners who need and receive long-term care (LTC). The importance of understanding the consequences of partner care receipt for psychological well-being among midlife and older individuals is broadly acknowledged (Bom et al., 2019; Verbakel et al., 2018). However, little research to date has compared well-being across different partner care arrangements, including individuals providing care to their ailing partner alone (“solo-care”), sharing care with, or outsourcing care, to informal or formal providers (Taylor et al., 2008). Rising LTC needs and the diversity of care arrangements in midlife and older European couples warrant a dyadic investigation of individuals’ well-being across the full spectrum of partner care arrangements.

Gender is an important moderator of the association between partner care arrangements and well-being, as it shapes responsibilities and expectations around partner care. Gender roles with respect to caregiving depend on the context in which care is embedded (Lewis, 1992). “Care contexts” encompass nations’ institutional, cultural, and structural elements that influence the responsibilities allocated to families, the state, and the market to meet the care needs of vulnerable adults (Leitner, 2003). While previous studies have documented gender differences in the association between partner care arrangements and well-being (Taylor et al., 2008), no study to date has explored how gender roles in relation to caregiving may moderate this association across different contexts. Our primary aim is to explore gender differences in individuals’ well-being by partner care arrangements across care contexts defined by four welfare regimes: Northern, Western, Southern, and Eastern Europe (Herlofson & Brandt, 2020).

Across Europe, recent reforms have aimed to curb rising LTC expenditures by reducing publicly funded or subsidized formal LTC, thus increasing the share of care responsibilities borne by informal caregivers (Fernandez et al., 2016). These changes may have consequences for the well-being of care recipients and their partners. Previous evidence suggests that the association between partner care provision and caregivers’ well-being depends on the availability of formal care options (Wagner & Brandt, 2018). However, to our knowledge, no study has comprehensively addressed contextual differences in individuals’ well-being across diverse partner care arrangements. Our secondary aim is to examine how differences in well-being between individuals whose partner is in informal and formal care arrangements vary across the four European contexts identified above.

This study bridges two bodies of caregiving literature toward a better understanding of the association between partner care and well-being. First, by describing gendered variation across Northern, Western, Southern, and Eastern Europe, we explore how gender roles in relation to caregiving moderate the association. Second, by describing differences in the association between informal and formal partner care arrangements across care contexts we hint at how the availability of alternative options to family care relates to the well-being of LTC recipients’ partners.

## Background

### Conceptualizing Gender Differences in Well-Being by Partner Care Arrangement

Differences in individuals’ well-being across partner care arrangements may reflect multiple concurrent factors, including care-induced stress, concordance in physical and emotional health between partners, and individual- and couple-level characteristics such as socioeconomic status and relationship quality. We posit that gender moderates the association between partner care arrangements and individuals’ well-being by acting upon normative expectations of primary caregiving roles within couples. Compared to their male partners, midlife and older women are more strongly socialized into caregiver—as opposed to “breadwinner”—roles, aligned with their lower life-course employment and earnings (Hank & Jürges, 2007). Women typically adopt more intensive care responsibilities for their partners (Pinquart & Sörensen, 2006), and may be subject to greater expectations to (solely) provide partner care than men (Calasanti & King, 2007).

The stress process model (Pearlin et al., 1990) provides a framework for understanding how different care arrangements may be associated with varying degrees of stress and in turn, how these may differentially shape well-being among women and men with ailing partners. Stressors arise from the care recipients’ illness, such as low cognitive functioning or behavioral problems. Such stressors are negatively associated with well-being among potential caregivers, both directly through increased intensity of care provision, and indirectly through intrapsychic strains related to the challenges of managing caregiving and other social responsibilities (Swinkels et al., 2019; Verbakel et al., 2018).

Within the stress process framework, *shared care* may improve well-being relative to *solo-care*, by providing caregivers with greater agency within their care situation (Pearlin et al., 1990), or by decreasing care intensity and subsequent exposure to psychological strain (Verbakel et al., 2018). However, *shared care* may present strain due to conflicts about care decisions (Wawrziczny et al., 2017) or low perceived quality of additional support (Juntunen et al., 2018). Regarding *outsourced* (formal or informal) partner *care*, the stress process model predicts higher well-being relative to *solo-* or *shared care*, due to relief from care-related stressors (Pearlin et al., 1990). Indeed, a large body of literature finds care provision to be linked with lower well-being, both in terms of associations (Pinquart & Sörensen, 2003) and causal effects (Bom et al., 2019).

Aligned with the stress process model, greater female caregiving responsibilities imply greater care-related stressors for women relative to men (Swinkels et al., 2019). Existing empirical evidence shows stronger negative effects of caregiving on well-being for women than men (Bom et al., 2019). Among older couples in the United States, *shared care* is associated with lower well-being than *solo-* or *outsourced care* for women than it is for men (Taylor et al., 2008). However, studies of Dutch and Finnish caregivers suggest that *shared care* is associated with lower well-being than *solo-care* for women and men alike (Juntunen et al., 2018; Swinkels et al., 2019). Research comparing *outsourced care* to other arrangements is inconclusive about gender differences (Taylor et al., 2008).

Gendered variations in well-being across partner care arrangements potentially reflect concordance in emotional well-being within couples. Empirical research on mental health concordance overwhelmingly suggests strong within-couple correlations of depressive symptoms and well-being (Meyler et al., 2007). *Shared* as well as *formal care* arrangements may reflect worse health and higher care needs of the care-receiving partner than *solo-*, *outsourced*, or *informal* care (Andersen & Newman, 2005). This suggests that *shared care* is related to lower well-being than *solo-* or *outsourced care*, while partners’ receipt of *formal* care may be related to lower well-being than *informal* arrangements for both members of the couple. A study on Mexican American married couples suggests that women are more vulnerable than men to negative emotional reactions to a partner’s poor well-being (Peek et al., 2006). Across contexts, however, there is clear evidence of mutual well-being influences for partners of both genders (Meyler et al., 2007).

Additionally, individual- and couple-level factors likely influence both an individual’s well-being and their partner’s care arrangements. Among the observable factors, partners’ health and socioeconomic status, age, and family characteristics (e.g., presence of adult children) have been identified as correlated both with partner care arrangements (Bertogg & Strauss, 2020) and well-being (Verbakel et al., 2018). Because these factors vary by gender, controlling for them is important when studying gender moderation in the association between partner care and well-being. Among the unobservable factors, personality traits and the quality of the relationship may correlate with one’s well-being as well as their partner’s care arrangement (Walker & Luszcz, 2009).

Overall, once covariates are controlled for, *shared care* may be associated with lower individual well-being than *solo-care*, due to greater care stressors and worse partner health. Moreover, women may be more susceptible than men to such care-related stressors (Verbakel et al., 2018). In turn, *solo-care* may be associated with lower well-being than *outsourced care*, as the latter entails complete relief from care stressors and lower partner care needs (Pearlin et al., 1990). In this case, the direction of gender moderation is ambiguous. As care provision is generally worse for women’s well-being than it is for men’s (Bom et al., 2019), the positive association between *outsourced care* (especially *informal*) and well-being may be stronger among women. Alternatively, the positive association between *outsourced care* (relative to *solo-* or *shared care*) and well-being may be stronger for men, as failing to fulfill one’s care obligations may be linked with lower well-being among women (Verbakel et al., 2018). Moreover, given that it is highly normative for women to provide partner care (Pinquart & Sörensen, 2006), women with preexisting lower well-being may select into *outsourced care* arrangements. Crucially, the extent and direction of gender moderation may depend on the context where care is embedded.

### The Role of the Care Context

Research has shown that care contexts influence gender roles in relation to caregiving as well as the availability and acceptability of formal alternatives to family care (Leitner, 2003). We follow previous literature on caregiving and care receipt (Carrieri et al., 2017; Herlofson & Brandt, 2020) in specifying four European geographic care contexts: North, West, South, and East. The Northern European “social-democratic” model is characterized by a universalistic, service-based approach to formal care provision, in which families are relieved of intensive care responsibilities (Esping-Andersen, 1999). The Western European “conservative-corporatist” approach combines high levels of formal care provision with explicit public support to family caregivers (Esping-Andersen, 1999; Leitner, 2003). In the Southern European “familistic” model the low provision of formal care services generates implicit reliance on family caregiving (Leitner, 2003). Finally, in the “post-socialist” model of Eastern Europe the continuation of universalistic approaches to care provision from the socialist legacy combined, in practice, with low provision and quality of services leaves the bulk of care responsibilities to families (Haggard & Kaufman, 2008).

Considering LTC indicators such as residential and home care expenditure and benefits, Northern and Western European countries typically provide greater institutional support to older individuals with disabilities compared to Southern and Eastern European countries (Leitner, 2003; Saraceno & Keck, 2010). Contextual differences in social policy approaches to care for older adults also influence the gendered division of roles regarding informal care and paid work (Saraceno & Keck, 2011; Schmid et al., 2012). Whereas the universalistic approach to LTC provision in Northern and Eastern Europe favors more equal gender division of roles (Haggard & Kaufman, 2008; Lewis, 1992), policies such as cash-for-care benefits and formal care infrastructure (or lack thereof) in Western and Southern European countries support or reinforce traditional gendered family care roles (Leitner, 2003; Lewis, 1992). While our study does not directly examine contextual LTC or normative indicators, the geographic classification captures predominant features that likely contribute to contextual variation in the gendered relationship between partner care arrangements and individuals’ well-being.

The stress process model does not directly offer predictions about the differential effect of *informal* as opposed to *formal* (*shared* or *outsourced*) partner care arrangements for individuals’ well-being. Cross-national studies suggest that greater contextual availability of formal support helps reduce the intensity of family care and care-related stressors, especially for women (Schmid et al., 2012; Wagner & Brandt, 2018). Verbakel et al. (2018) find that, in the Netherlands, *formal* support is related to higher caregivers’ well-being by reducing the intensity of care. At the same time, *informal* partner care arrangements (such as care from adult children) may enhance well-being by reinforcing social support for both partners (Berkman et al., 2000). Across contexts, the greater availability of formal care in Northern and Western—relative to Southern and Eastern—Europe may partly counteract lower well-being associated with the poor health of partners in *formal* care, reducing differences in well-being among individuals with partners in *formal* and *informal* (*shared* or *outsourced*) care arrangements.

Research on gender differences in the association between care provision and well-being suggests that female caregivers are more vulnerable to poor well-being outcomes in countries with strong social preferences for female family care (Brenna & Di Novi, 2016; Ruppanner & Bostean, 2014), but no study to date has examined differences across the partner care spectrum. We expect that any gender differences in the association between partner care arrangements and well-being will be emphasized in Western and Southern (relative to Eastern and Northern) Europe.

### Summary of Hypotheses

Based on the above discussion we provide three sets of hypotheses regarding gender and contextual variation in the associations of partner care arrangements with individuals’ well-being.

- H1: *Shared care* has a negative association with well-being compared to *solo-* or *outsourced care*, and
  ◦ H1a. The negative association is more pronounced for women than men;
  ◦ H1b. Gender differences are more pronounced in Western and Southern rather than Northern and Eastern Europe.
- H2: *Outsourced care* has a positive association with well-being compared to *solo-* or *shared care*, and
  ◦ H2a. The positive association is more pronounced for men than women;
  ◦ H2b. Gender differences are more pronounced in Western and Southern rather than Northern and Eastern Europe.
- H3: *Formal* (*shared* or *outsourced*) *care* has a negative association with well-being compared to *informal* (*shared* or *outsourced*) *care*, and
  ◦ H3a. The negative association is more pronounced in Southern and Eastern rather than Northern and Western Europe.

## Method

### Data and Sample Selection

We use data from the sixth wave of the Survey of Health, Ageing and Retirement in Europe (SHARE, version 7.0.0) collected in 2015 in 17 European countries (Börsch-Supan et al., 2013). SHARE is representative of the community-dwelling population aged 50+. At the time of writing, Wave 6 is the most recent regular SHARE wave that distinguishes personal care (i.e., help with tasks such as bathing, eating, and getting in and out of bed) from informal help with household chores or paperwork from people outside the household (Bertogg & Strauss, 2020). While attrition is present and varies across countries, retention rates to Wave 6 are generally high, and 10 countries have refreshment or baseline samples at Wave 6, enhancing representativeness (Bergmann et al., 2019).

We are interested in understanding how different partner care arrangements are associated with individuals’ life satisfaction and depressive symptoms. Our reference sample includes all couples where one partner is physically impaired (i.e., has limitations in activities of daily living, ADLs) and receives personal care. We closely follow the study by Bertogg and Strauss (2020) for the specification of the analytic sample of couples. SHARE Wave 6 has information on 49,115 respondents aged 50+ who live with their partner. Of these, we limit the sample to cases where both partners are interviewed (43,088 individuals in 21,544 couples), excluding 6,027 individuals whose cohabiting partner lives in an institution (*n* = 106) or is unavailable/refuses to participate in the survey (*n* = 5,924). Aligned with our research question, we restrict the sample to couples where both partners are aged 50+, and one receives personal care (4,238 observations). As 94% of couples in our sample are “legally married,” we do not distinguish between married and cohabiting couples. We match data from both partners so that each observation carries information about the “individual” (i.e., the person whose partner receives care) and the “partner” (i.e., the care recipient). Our final analytic sample is restricted to couples for which neither partner has missing values on any variable of interest. This consists of 3,465 observations, of which 1,842 refer to couples where the “individual” is a woman and the care-receiving “partner” is a man, and 1,623 refer to couples where the “individual” is a man and the care-receiving “partner” is a woman. Our analytic sample excludes approximately 18% of eligible couples due to missing observations on variables of interest. As most missing data come from our outcome (i.e., well-being) measures, multiple imputation was not advisable (Carpenter & Kenward, 2013). As Supplementary Table 1 shows, individuals (I) in the final analytic sample (*n* = 3,465) are slightly “healthier” than those in the eligible sample (*n* = 4,238), with fewer ADL/instrumental ADL (IADL) limitations and better self-rated health. However, the mean values of all variables do not differ significantly between the eligible and analytic sample.

### Measures

#### Outcomes

We use two well-being measures, life satisfaction and depressive symptoms, that measure different dimensions of well-being (Vanhoutte, 2014). Life satisfaction provides an overall evaluation of life across multiple domains and according to individual goals. It is a comparably stable cognitive self-assessment of general well-being (George, 2010; see Diener et al., 2018 for a detailed review) measured by a question asking respondents “on a scale from 0 to 10 where 0 means completely dissatisfied and 10 means completely satisfied, how satisfied are you with your life?” The single-item measure has been validated across contexts, and yields similar validity and reliability as multi-item scales (George, 2010). We treat it as an interval continuous variable ranging from 0 to 10 (low to high satisfaction). Depression captures negative emotional states, and it may be related to—but is distinct from—life satisfaction (Vanhoutte, 2014). Depressive symptoms are measured using the EURO-D 12-item scale (Prince et al., 1999). We treat this scale as continuous ranging from 0 to 12, and we reverse-code it such that values go from 0 (severely depressed) to 12 (no depressive symptoms). Similar to previous research (Kaschowitz & Brandt, 2017), reverse-coding is applied to ease interpretation of the results, so that higher scores indicate higher well-being for both outcomes.

#### Care arrangements

We follow Bertogg and Strauss (2020) to generate our main explanatory variable of interest, partner care arrangement. For simplicity, within each couple, we identify the “individual” as I and their care-receiving “partner” as P. We code our explanatory variable as follows: If I reports providing care for P, or P reports receiving care from I, we code this as partner care. We then distinguish among: (a) *solo-care*: P only receives care from I; (b) *shared informal care*: P receives care from I and other informal caregivers, but no formal care; (c) *shared formal care*: P receives care from I and formal providers, regardless of whether P additionally receives informal care from other carers. In our sample, about 38% of Ps receiving *shared formal* care also receive additional informal care. If I does not report providing care to P, and P does not report receiving care from I, we identify this as *outsourced care*. We then distinguish between (d) *outsourced informal care*: P only receives care from informal carers who are not I; and (e) *outsourced formal care*: P receives care from formal providers and not from I, regardless of whether P also receives care from informal carers. In our sample, 32% of Ps receiving *outsourced formal* care additionally receive care from other informal providers. Our variable only differs from the Bertogg and Strauss (2020) specification in that we use information from both I and P for the definition of partner care. Table 1 describes the survey questions used to derive the care arrangement variable. In 81.7% of cases (*n* = 2,831), couple’s reports of caregiving (I) and care receipt (P) are consistent. Inconsistencies are mainly related to P not reporting care receipt from a partner while I reports caregiving (14.7%), rather than the inverse (3.6%). To the extent that inconsistent cases are related to poor relationship quality or poor cognitive health, they may bias our estimates of the associations between partner care arrangements and well-being. Reassuringly, when replicating the analyses for the sample of couples with consistent care reports, the results remain substantively the same (as shown in Supplementary Figures 1 and 2).

**Table 1.** Survey Questions Used for the Derivation of the Care Arrangement Variable

	Solo-care	Shared informal	Shared formal	Outsourced informal	Outsourced formal
**Survey questions to partner (P):**					
** Sp021:** “Who helps you with personal care in your household?”: **spouse/partner**	✓	✓	✓		
** Sp021:** “Who helps you with personal care in your household?”: **any informal carer (relatives, friends) except spouse/partner**		✓	*	✓	*
** Sp021:** “Who helps you with personal care in your household?”: **therapist or other professional helper, home health care provider**			✓		✓
** Sp003/Sp004:** “which family member from outside the household, friend, or neighbor has helped you in the last 12 months?”: **any informal carer (relatives, friends, neighbors)**; “which type of help has this person provided?”: **personal care**		✓	*	✓	*
** Sp003/Sp004:** “which family member from outside the household, friend, or neighbor has helped you in the last 12 months?”: **therapist or other professional helper, home health care provider**; “which type of help has this person provided?”: **personal care**			✓		✓
** Hc127d1:** “during the last 12 months, did you receive in your own home any professional or paid services due to a physical, mental, emotional, or memory problem?”: **help with personal care**			✓		✓
**Survey questions to individual (I):**					
** Sp019d1:** Is there someone living in this household whom you have helped regularly during the last 12 months with personal care, such as washing, getting out of bed, or dressing?”: **spouse/partner**	✓	✓	✓		

*Notes*: ✓ indicates classification into corresponding care type depends on positive response.

* indicates classification into corresponding care type does not depend on positive/negative response.

#### Control variables

We control for characteristics of I and P, as well as couple-level characteristics that may correlate with both I’s well-being and P’s receipt of care. For I, we control for age, educational attainment, work status, presence of ADL or IADL limitations, poor self-rated health, and the number of social activities performed at least monthly, including voluntary work, educational or training courses, sport or social clubs, political organization, and playing cards or games. We control extensively for P’s health status, including number of ADL and IADL limitations; self-rated health; number of doctor-diagnosed conditions; and cognitive function. At the couple level, we control for equivalized couple-level income, household financial wealth, home ownership, and parental status (indicating whether the couple has children and, if so, whether any child lives in the household). All models include controls for country context by including a dummy variable for each country. Supplementary Table 2 shows the detailed coding of all the control variables in the study.

### Statistical Analysis

We use linear regression models to study the associations between P’s care arrangement and I’s well-being. For each outcome, we start from an unadjusted model in which the outcome is regressed on P’s care arrangement only including country fixed-effects (dummies). We then add I’s, P’s, and couple characteristics as covariates. The nature of our sample (with each observation containing information about both the individual and their partner) allows us to control extensively for the P’s health, which partly accounts for well-being concordance within couples (Meyler et al., 2007). For each outcome, we compare results from the unadjusted and fully adjusted models. We test for gender moderation by interacting P’s care arrangements with I’s gender. This is generally appropriate when studying well-being-related outcomes, due to gender differences in overall levels and determinants of well-being (Schmitz & Brandt, 2019).

We test for heterogeneity in the gendered associations between care arrangements and well-being outcomes across four care contexts, which broadly reflect the typologies identified above (Esping-Andersen, 1999; Haggard & Kaufman, 2008):

- Northern Europe: Denmark and Sweden
- Western Europe: Austria, Belgium, France, Germany, Luxembourg, and Switzerland
- Southern Europe: Greece, Italy, Portugal, and Spain
- Eastern Europe: Croatia, Czech Republic, Estonia, Poland, and Slovenia

We replicate the fully adjusted models separately by care context, and test for gender moderation using interactions, as above. We estimate heteroscedasticity-robust standard errors for all models. We use Stata version 16.

## Results

### Descriptive Sample Characteristics

Table 2 shows the percentage of couples in each care arrangement with corresponding mean values of I’s well-being, separately by care context and I’s gender. Corresponding *p* values for the significance of gender differences in care arrangements and well-being are also reported. As previously observed (Bertogg & Strauss, 2020), women are significantly more likely than men to be *solo-caregivers* (58% vs 52%), while men are significantly more likely than women to *outsource* partner *care*, especially to informal caregivers. Gender differences in care arrangements are largest and statistically significant in Southern, and smallest and not significant in Northern European countries: 22% of Southern European men in our sample *outsource* care to *informal* caregivers, compared to 11% of Southern European women. Women with partners in *shared formal* care have, on average, the lowest levels of well-being. By contrast, women *outsourcing* partner care (either formally or informally) have the highest average well-being, which may reflect relief from care as well as better partner health. Among men, *shared informal* care is associated with the lowest life satisfaction, while *outsourced informal* care with the highest life satisfaction. However, men in *outsourced formal* care have the lowest well-being scores in terms of higher number of depressive symptoms.

**Table 2.** Sample Characteristics by Care Context and Individual’s (I) Gender, With *p* Values for Chi-Squared and *t*-Tests of Gender Differences

	Total			North			West			South			East		
I’s gender	Female	Male	*p*(diff)	Female	Male	*p*(diff)	Female	Male	*p*(diff)	Female	Male	*p*(diff)	Female	Male	*p*(diff)
Solo-care (%)	**58.6**	**52.2**	**<.001**	**62.2**	**58.3**	**.291**	**56.2**	**53.4**	**.002**	**59.3**	**48.1**	**<.001**	**59.3**	**53.2**	**.030**
Mean life satisfaction (0–10)	7.19	7.38	.038	8.43	8.57	.633	7.65	7.64	.926	6.91	6.99	.616	6.80	7.12	.070
Mean reverse-coded EURO-D (0–12)	8.49	9.30	<.001	9.43	10.1	.042	8.81	9.62	<.001	7.98	8.50	.051	8.42	9.48	<.001
Shared informal (%)	**13.4**	**12.5**	**<.001**	**4.7**	**5.0**	**.291**	**7.0**	**6.8**	**.002**	**13.9**	**11.9**	**<.001**	**20.1**	**20.4**	**.030**
Mean life satisfaction (0–10)	7.10	7.04	.785	8.50	8.33	.852	7.16	7.72	.151	7.10	6.79	.371	6.98	6.86	.614
Mean reverse-coded EURO-D (0–12)	8.21	9.17	<.001	7.83	9.83	.166	8.61	9.44	.092	7.94	8.79	.087	8.35	9.20	.011
Shared formal (%)	**13.2**	**11.7**	**<.001**	**16.5**	**15.8**	**.291**	**21.6**	**15.7**	**.002**	**10.1**	**11.4**	**<.001**	**8.0**	**6.9**	**.030**
Mean life satisfaction (0–10)	6.98	7.37	.030	7.79	8.06	.590	7.41	7.91	.032	6.02	7.08	.008	6.77	6.44	.330
Mean reverse-coded EURO-D (0–12)	8.10	9.08	<.001	8.53	9.38	.200	8.64	9.44	.013	6.30	8.22	.001	8.42	9.53	.059
Outsourced informal (%)	**9.3**	**16.9**	**<.001**	**7.9**	**15.0**	**.291**	**7.2**	**13.6**	**.002**	**10.1**	**21.8**	**<.001**	**10.7**	**16.4**	**.030**
Mean life satisfaction (0–10)	7.43	7.68	.149	9.38	9.35	.954	8.33	8.16	.640	6.43	7.58	<.001	7.40	6.88	.103
Mean reverse-coded EURO-D (0–12)	8.74	9.47	.004	10.0	11.1	.077	9.79	10.0	.500	7.51	8.86	.009	8.94	9.40	.320
Outsourced formal (%)	**5.5**	**6.7**	**<.001**	**8.7**	**5.8**	**.291**	**8.0**	**10.5**	**.002**	**6.7**	**6.9**	**<.001**	**2.0**	**3.1**	**.030**
Mean life satisfaction (0–10)	7.58	7.52	.702	8.73	7.71	.202	7.85	8.12	.380	7.03	6.81	.623	7.00	7.38	.673
Mean reverse-coded EURO-D (0–12)	8.32	8.95	.061	9.64	9.29	.660	8.33	9.17	.072	8.03	8.37	.654	8.33	10.4	.018
*N* observations	**1,842**	**1,623**		**127**	**120**		**541**	**515**		**526**	**464**		**648**	**524**	

*Note*: SHARE = Survey of Health, Ageing and Retirement in Europe. Values in bold indicate percentages in each partner care arrangement group by gender, and corresponding p-values for gender differences.

*Source*: SHARE Wave 6 (2015).

Supplementary Tables 3 and 4 report descriptive statistics for all the covariates by I’s gender, for women and men, respectively. As expected, P’s health correlates with the type of care arrangement, with partners in *shared formal* care displaying the worst physical, cognitive, and self-rated health, followed by partners in *shared informal* and *outsourced formal* care. Ps in *outsourced informal* care have, on average, the fewest health limitations. Similarly, individuals *outsourcing* partner care to *formal* (not *informal*) providers have the worst health status among all groups.

### Multivariate Results

**Figure 1.**
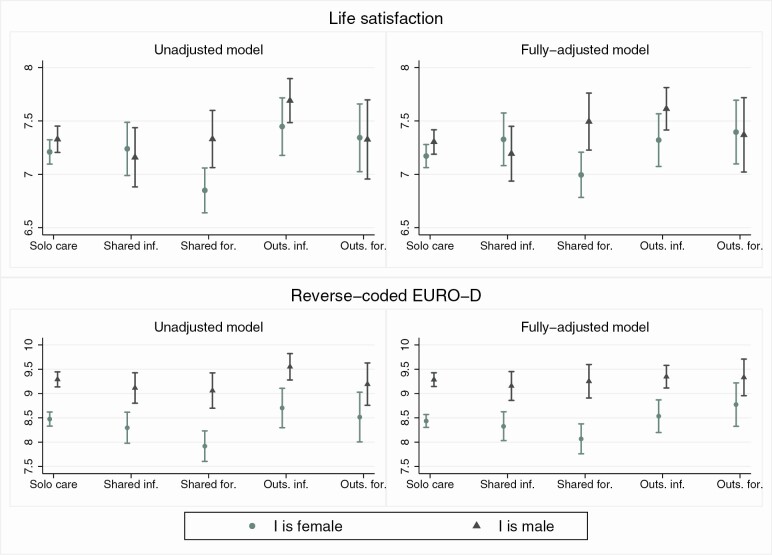
Predicted scores for life satisfaction and reverse-coded EURO-D scores by gender (*N* = 3,465). *Source*: SHARE Wave 6 (2015). SHARE = Survey of Health, Ageing and Retirement in Europe.

Figure 1 shows the average predicted scores for life satisfaction and reverse-coded EURO-D (with higher scores indicating fewer depressive symptoms) associated with different care arrangements, separately by unadjusted and fully adjusted models. The results come from linear regression models that include interactions between P’s care arrangement and I’s gender. Predicted scores are displayed separately by I’s gender, along with their 95% confidence intervals. The confidence intervals indicate whether the associations are significantly different by gender, and whether they are significantly different across care arrangements within gender (i.e., differences are statistically significant if the confidence intervals do not overlap). Supplementary Table 5 provides the corresponding full-model regression coefficients.

Overall, life satisfaction scores do not differ by gender, but women report more depressive symptoms, confirming previous findings (Schmitz & Brandt, 2019). In line with H1a, we observe a significant difference in the association between *shared care* and well-being by gender, with *shared formal care* associated with significantly lower well-being for women than for men (i.e., lower life satisfaction and more depressive symptoms).

**Figure 2.**
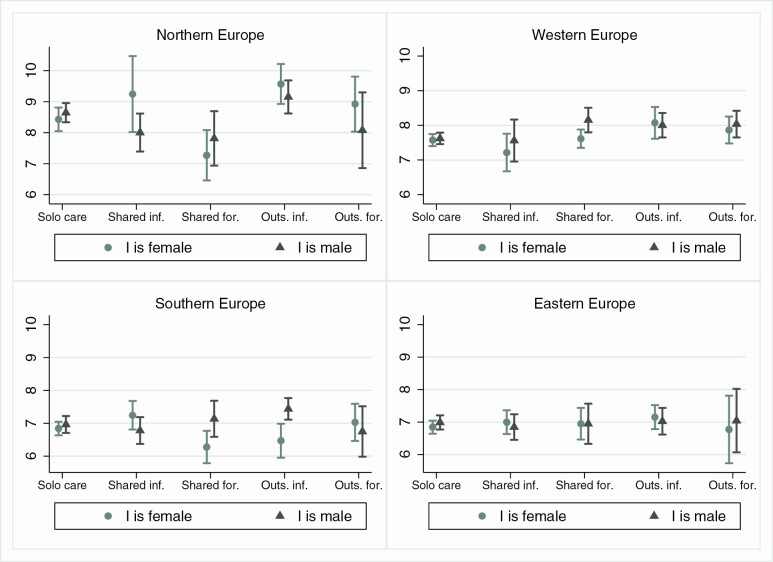
Predicted scores for life satisfaction by I’s gender and care context (fully adjusted models). *Source*: SHARE Wave 6 (2015). SHARE = Survey of Health, Ageing and Retirement in Europe.

**Figure 3.**
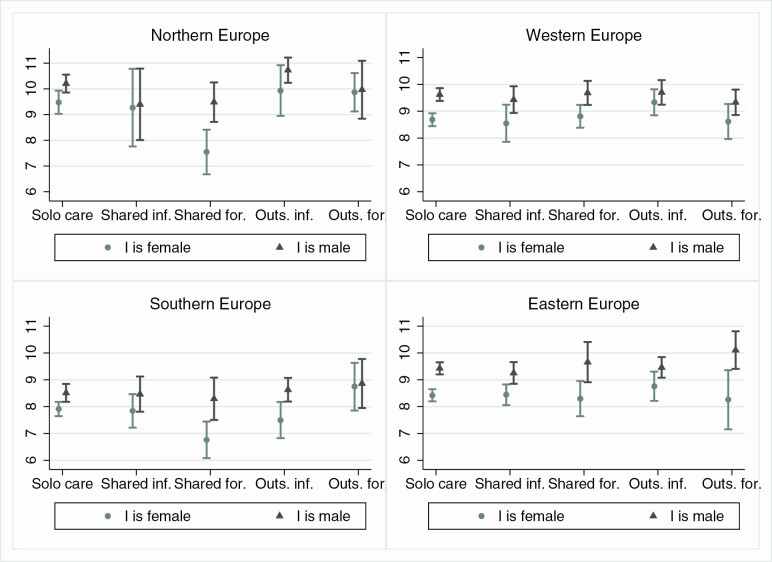
Predicted scores for reverse-coded EURO-D by I’s gender and care context (fully adjusted models). *Source*: SHARE Wave 6 (2015). SHARE = Survey of Health, Ageing and Retirement in Europe.

In Figures 2 and 3 we explore the potential contextual drivers of the association in terms of strength of gender roles and availability of formal care services (Supplementary Tables 6 and 7 provide complete regression coefficients). Aligned with H1b, gender differences in the association between *shared formal* care and well-being are only detected in Southern and Western Europe. However, while in Southern Europe women have significantly lower well-being in *shared formal* (compared to *solo-*, *shared informal*, and *outsourced formal* care arrangements), the association is not statistically significant in Western Europe, where the gender difference is driven by the fact that men have generally higher well-being when having a partner in *shared formal* care. In Northern Europe, both men and women with partners in *shared formal care* have lower well-being outcomes than in other care arrangements; in Eastern Europe, no statistically relevant differences in well-being by care arrangement emerge for either gender.

The second result that emerges from the cross-context aggregate associations in Figure 1 is that for men, but not women, *outsourced informal* care is associated with significantly higher life satisfaction than other care arrangements, which is in line with H2a. Figure 2 suggests that *outsourced informal* care is associated with higher life satisfaction than *solo-* and *shared formal* care for men in Northern, Southern, and Western Europe. We find that the same is true for Northern and Western European women, with no gender differences in these contexts. Again, we find a significant gender difference in Southern Europe, which partially supports H2b of greater gender differences in contexts with greater reliance on female family care, although the same does not hold in Western Europe.

Finally, in line with H3, we find that *shared informal* care is associated with higher well-being than *shared formal* care. As this result is mainly driven by Northern and Southern Europe, H3a that differences between *formal* and *informal* care arrangements would be greater in family-based (as opposed to service-based) care contexts is not supported.

## Discussion

This is the first study to examine gender and contextual differences in well-being among potential partner caregivers across a diverse range of partner care arrangements, differentiating by care from *informal* and *formal* caregivers and by whether partners *share* or *outsource* caregiving. Our dyadic analysis allows us to control for the characteristics of both partners, which is important given health and well-being concordance within couples (Meyler et al., 2007).

Our first set of hypotheses is strongly confirmed. *Shared formal* care is associated with lower well-being for women than men (H1a). Within the stress process model (Pearlin et al., 1990), this suggests that gendered responsibilities to provide partner care may generate unique stressors for women when *sharing care* for their partner with others (Pinquart & Sörensen, 2006). This result also hints at the gendered nature of well-being concordance within couples, as women potentially suffer relatively more than men from having a partner in poorer health (Peek et al., 2006). Furthermore, gender differences are only detected in Southern and Western European countries (H1b). However, a female “penalty” in well-being for s*hared formal* care—as opposed to other arrangements—is only present in Southern Europe. This suggests that in care contexts with strong normative expectations for women to provide care and low formal care availability, *shared formal* partner care potentially creates additional stressors for women. These may reflect social stigma of not fulfilling socially expected roles as the sole caregiver for their partner (Ruppanner & Bostean, 2014).

Our second set of theoretical hypotheses is also confirmed. *Outsourced* partner care is generally associated with higher well-being than other arrangements, suggesting relief from care stressors within the stress process framework (Pearlin et al., 1990). We observe gender differences in Southern Europe, where *outsourced informal care* is associated with lower well-being for women than other arrangements (H2a, H2b). As Verbakel et al. (2018) have hypothesized, women in *outsourced care* may experience lower well-being due to perceived nonfulfillment of their caregiving roles. Our findings suggest this is potentially relevant in Southern Europe, given strong gender norms related to women’s caregiving (Lewis, 1992; Saraceno & Keck, 2011).

Finally, our hypothesis set H3 is not fully supported, as we find no consistent differences in individuals’ well-being between *informal* and *formal* partner care arrangements across contexts. We find *outsourced informal* care to be associated with higher well-being for men. Such an arrangement may expand men’s social network through increased contact with adult children who provide care for the ailing partner. From this perspective, enhancing community services to expand men’s social and support networks may help alleviate the strains derived from having an ailing partner, and protect well-being. For women, the results suggest that *shared formal* care is linked with lower well-being than *shared informal* care, which may be attributable to inefficiencies in coordinating care with formal providers (Lamura et al., 2008). Policies aimed at supporting family caregivers should be sensitive to the coordination of care networks between domiciliary formal and informal providers, especially in contexts where the former are not widely available.

This study has several limitations. Our use of cross-sectional data implies that we cannot relate changes in partner care receipt to changes in well-being, or discern the temporal order of events, which limits our ability to assess underlying causal mechanisms. Similarly, we cannot distinguish associations based on the duration of care arrangements. Length of care indicates the chronicity of the stressors of the caregiver, which may substantially affect their well-being (Pearlin et al., 1990).

The availability of formal LTC, caregiver support measures, as well as gender and family norms vary widely across countries (Saraceno & Keck, 2010, 2011). Our study does not examine macro-level indicators (e.g., public LTC expenditure), and cannot suggest specific explanations for contextual differences in the gendered associations between partner care arrangements and well-being. With more data on a greater number of countries, one could use multilevel modeling to test for macro–micro interactions in the associations of care arrangements with well-being. Because SHARE is only representative of the community-dwelling population, our sample is restricted to couples where both partners are healthy enough to take part in the SHARE interview, and live at home rather than in care homes or institutions, the presence of which varies widely across countries (Saraceno & Keck, 2010). By excluding the most severely impaired partners, we potentially underestimate any negative association between providing partner care and individual’s well-being.

Due to data limitations, we do not test any individual-level mechanisms that may shed light on the gendered patterns of well-being by partner care arrangements. Although we control extensively for partner’s health, our analysis would benefit from including additional indicators such as marital relationship quality (Wawrziczny et al., 2017). Moreover, the exclusion of couples where one partner refuses or is unavailable to participate in the survey may generate biases if partner nonresponse is related to relationship quality. We cannot examine hours of care provision, which limits our ability to account for the extent of *shared care* responsibilities. Due to small sample sizes, we are unable to differentiate relationships among potential informal carers with whom partners *share* (or *outsource*) care (e.g., adult children). Future research may fruitfully add these dimensions, especially as we suspect that *outsourced informal* care may be particularly beneficial for men if provided by adult children.

Our findings provide a useful frame of reference for future studies to disentangle the complex associations between partner care arrangements and well-being. Current LTC reforms in Europe are increasingly shifting care responsibilities to family members (Fernandez et al., 2016), and partners are often the first port of call for those with care needs. Within care contexts, it is important for researchers and policymakers to consider the diversity of potential care arrangements and the role of gender when studying the well-being of potential caregivers. Beyond single-country contexts, cross-national differences in gendered family norms and care preferences, as well as in the availability of formal care options, need to be taken into account. Future research investigating the macro-level moderators of the association between partners’ care arrangements and well-being may elucidate contextualized and gender-sensitive policy responses to LTC planning that promote middle-aged and older adults’ well-being.

## Supplementary Material

gbab209_suppl_Supplementary_Material
