# Supplementary material for: Partner Care Arrangements and Well-Being in Mid- and Later Life: The Role of Gender Across Care Contexts
Source: J Gerontol B Psychol Sci Soc Sci. 2021 Nov 9;77(2):435–45. doi: 10.1093/geronb/gbab209 (PMC8824554; doi:10.1093/geronb/gbab209)
Supplement: gbab209_suppl_Supplementary_Material [file gbab209_suppl_supplementary_material.pdf]

Supplementary tables and figures

Supplementary table 1. Analysis of missingness of all relevant variables in the sample

	n. (%) missing of eligible sample (n = 4,238)	Eligible sample distribution (obs. with non-missing values)	Analytic sample distribution (n = 3,465)
**Individual outcomes**			
Life satisfaction (0-10)	347 (8.19 %)	7.26	7.28
Reverse-coded EURO-D (0-12)	364 (8.59 %)	8.76	8.81
**Individual characteristics**			
Gender: % female	0 (0.00 %)	52.31	53.47
Age	0 (0.00 %)	70.63	70.18
Education: % low	80 (1.89 %)	51.80	50.41
% intermediate		32.92	34.12
% high		15.27	15.47
% employed or self-employed	11 (0.26 %)	12.30	12.67
% 1+ ADL limitations	4 (0.09 %)	22.25	18.84
% 1+ IADL limitations	4 (0.09 %)	30.70	26.75
% poor self-rated health	5 (0.12 %)	19.89	16.29
Social activities: % none	0 (0.00 %)	65.01	61.80
% one activity		22.65	24.54
% two or more activities		12.34	13.66
**Partner characteristics**			
ADL limitations (n)	4 (0.09 %)	1.46	1.46
IADL limitations (n)	4 (0.09 %)	1.90	1.86
% poor self-rated health	3 (0.07 %)	42.17	42.39
Diagnosed conditions (n)	2 (0.05 %)	2.22	2.21
% low cognitive function	0 (0.00 %)	9.86	9.86
**Couple characteristics**			
Fin. wealth: 1st quintile gr.	59 (1.39 %)	24.96	24.28
2nd		21.80	21.39
3rd		19.86	20.29
4th		18.11	18.42
5th		15.27	15.61
Income: 1st quintile group	89 (2.10 %)	18.05	17.63
2nd		21.62	21.76
3rd		24.87	24.79
4th		21.14	21.54
5th		14.32	14.28
% home ownership	8 (0.19 %)	76.50	76.93
Parent status: % childless	49 (1.16 %)	6.61	6.26
% with children outside hh		69.32	69.76
% with co-resident children		24.06	23.97

Source: SHARE wave 6 (2015).

Supplementary table 2. Coding of all the control variables in the study

Variable	Coding
**Individual (I) characteristics**	
Age	Years (min = 50, max = 95)
Education	1 = low (up to lower secondary, ISCED 0-2); 2 = intermediate (upper secondary, ISCED 3-4); 3 = high (tertiary, ISCED 5-6)
Work status	0 = not employed or self-employed; 1 = employed or self-employed
ADL limitations	0 = no limitations with ADLs; 1 = one or more ADL limitations
IADL limitations	0 = no limitations with IADLs; 1 = one or more IADL limitations
Poor self-rated health	0 = not in poor health; 1 = “poor” self-reported health
Social activities	0 = no social activities performed at least monthly in the last year; 1 = one social activity performed at least monthly in the last year; 2 = two or more social activities performed at least monthly in the last year.
**Partner (P) characteristics**	
ADL limitations	Number of ADL limitations (min = 0, max = 6)
IADL limitations	Number of IADL limitations (min = 0, max = 7)
Poor self-rated health	0 = not in poor health; 1 = “poor” self-reported health
Diagnosed conditions	Number of diagnosed conditions (min = 0, max = 11)
Low cognitive function	0 = not low cognitive function; 1 = low cognitive function: respondent has either a low memory score (i.e. fewer than 8 out of 20 words recalled) or a low time orientation score (i.e. two or more mistakes in identifying day of the week, date, month and year).
**Couple characteristics**	
Financial wealth	Natural logarithm/inverse hyperbolic sine of equivalised household financial wealth, in Purchasing Power Parity (PPP) Euros. Note: split into five equal quintile groups for descriptive statistics.
Income	Natural logarithm/inverse hyperbolic sine of equivalised couple income, in Purchasing Power Parity (PPP) Euros. Note: split into five equal quintile groups for descriptive statistics.
Home ownership	0 = no one in the household owns the home where the couple live; 1 = someone in the household owns the home where the couple live.
Parent status	0 = childless; 1 = with children, all living outside the household; 2 = with children, and at least one co-resident child
**Country**	One dummy variable for each of the following countries: Austria, Belgium, Croatia, Czech Republic, Denmark, Estonia, France, Germany, Greece, Italy, Luxembourg, Poland, Portugal, Slovenia, Spain, Sweden, and Switzerland.

Supplementary table 3. Descriptive statistics for the analytic sample of women aged 50+ by care arrangement for partner

Subsample of women 50+	Total	Solo caregiving	Shared informally	Shared formally	Outsourced informally	Outsourced formally
**Individual outcomes**						
Life satisfaction (0-10)	7.19	7.19	7.10	6.98	7.43	7.58
Reverse-coded EURO-D (0-12)	8.41	8.49	8.21	8.10	8.74	8.32
**Individual characteristics**						
Age	69.25	68.42	69.69	71.59	67.41	74.02
Education: % low	53.78	53.63	60.33	49.18	46.95	61.76
% intermediate	33.61	33.87	32.64	35.66	35.37	25.49
% high	12.61	12.50	7.02	15.16	17.68	12.75
% Employed or self-employed	11.31	11.75	7.05	11.93	17.28	5.88
% 1+ ADL limitations	18.38	19.87	12.86	13.58	19.14	26.47
% 1+ IADL limitations	29.30	29.80	24.07	28.81	27.16	41.18
% poor self-rated health	15.65	15.57	14.52	11.93	16.67	26.47
Social activities: % none	63.32	61.70	72.20	64.20	62.96	62.75
% one activity	23.12	25.41	19.09	19.34	20.99	21.57
% two or more activities	13.26	12.89	8.71	16.46	16.05	15.69
**Partner characteristics**						
ADL limitations (n)	1.58	1.24	2.42	3.03	0.47	1.44
IADL limitations (n)	2.01	1.58	2.99	3.60	0.90	2.07
% poor self-rated health	45.85	42.88	62.66	60.91	23.46	36.27
Diagnosed conditions (n)	2.24	2.19	2.56	2.51	1.72	2.21
% low cognitive function	11.20	9.46	13.28	13.99	10.49	18.63
**Couple characteristics**						
Fin. wealth: 1st quintile group	23.83	21.28	27.27	25.00	29.27	30.39
2nd	21.72	22.90	21.49	23.36	12.80	20.59
3rd	20.72	20.90	19.83	20.90	20.12	21.57
4th	19.11	18.89	17.77	18.44	21.95	21.57
5th	14.61	16.03	13.64	12.30	15.85	5.88
Income: 1st quintile group	17.22	16.79	18.60	15.57	17.68	21.57
2nd	22.11	21.85	23.97	25.00	15.85	23.53
3rd	25.22	25.00	25.21	27.46	20.73	29.41
4th	22.17	23.00	22.31	20.49	25.00	12.75
5th	13.28	13.36	9.92	11.48	20.73	12.75
% home ownership	77.38	78.80	80.08	70.37	77.16	73.53
Parent status: % childless	6.21	7.48	2.93	7.82	2.48	2.94
% with children outside hh	71.14	70.37	71.97	74.90	65.22	77.45
% with co-resident children	22.65	22.15	25.10	17.28	32.30	19.61
**N. observations**	**1,842**	**1,079**	**247**	**243**	**171**	**102**
**Sample percentage**		**58.58**	**13.41**	**13.19**	**9.28**	**5.54**

Source: SHARE wave 6 (2015).

Supplementary table 4. Descriptive statistics for the analytic sample of men aged 50+ by care arrangement for partner

**Subsample of men 50+**	**Total**	**Solo caregiving**	**Shared informally**	**Shared formally**	**Outsourced informally**	**Outsourced formally**
**Individual outcomes**						
Life satisfaction (0-10)	7.39	7.38	7.04	7.37	7.68	7.52
Reverse-coded EURO-D (0-12)	9.26	9.30	9.17	9.08	9.47	8.95
**Individual characteristics**						
Age	72.28	71.23	72.56	76.76	69.97	76.23
Education: % low	47.89	46.19	50.00	45.77	51.42	52.25
% intermediate	33.90	36.68	33.68	35.82	25.91	28.73
% high	18.22	17.13	16.32	18.41	22.67	18.92
% employed or self-employed	11.39	11.17	8.42	7.46	17.81	10.81
% 1+ ADL limitations	19.84	22.84	16.32	14.93	15.38	23.42
% 1+ IADL limitations	24.53	26.02	21.05	22.39	19.84	34.23
% poor self-rated health	17.31	17.51	17.37	16.42	16.60	18.92
Social activities: % none	60.18	57.74	59.47	64.68	63.16	63.96
% one activity	25.63	25.38	27.37	25.87	24.29	27.03
% two or more activities	14.18	16.88	13.16	9.45	12.55	9.01
**Partner characteristics**						
ADL limitations (n)	1.39	1.05	2.03	2.92	0.65	1.57
IADL limitations (n)	1.77	1.29	2.44	3.76	0.94	2.24
% poor self-rated health	39.23	32.99	53.68	63.18	28.74	38.74
Diagnosed conditions (n)	2.23	2.14	2.58	2.73	1.83	2.23
% low cognitive function	8.85	7.23	8.95	14.43	8.50	10.81
**Couple characteristics**						
Fin. wealth: 1st quintile group	23.86	22.78	21.88	20.90	28.79	28.95
2nd	20.86	20.53	20.31	25.37	18.68	21.05
3rd	19.71	20.15	16.67	19.90	19.46	21.93
4th	18.36	19.02	25.52	16.92	15.18	11.40
5th	17.21	17.52	15.62	16.92	17.90	16.67
Income: 1st quintile group	17.15	15.39	16.67	14.93	23.35	20.18
2nd	21.63	21.53	20.83	24.38	20.62	21.05
3rd	24.76	27.16	26.04	23.88	16.73	25.44
4th	21.05	21.28	20.83	20.40	22.57	17,54
5th	15.42	14.64	15.62	16.42	16.73	15.79
% home ownership	77.29	77.03	81.05	72.14	80.16	75.68
Parent status: % childless	6.25	6.07	6.77	9.55	4.71	4.39
% with children outside hh	69.95	73.58	61.46	77.89	56.86	74.56
% with co-resident children	23.79	20.35	31.77	12.56	38.43	21.05
**N. observations**	**1,623**	**847**	**203**	**189**	**275**	**109**
**Sample percentage**		**52.19**	**12.51**	**11.65**	**16.94**	**6.72**

Source: SHARE wave 6 (2015).

Supplementary table 5. Figure 1: corresponding regression coefficients with robust standard errors in parentheses

	**Life satisfaction**		**Reverse-coded EURO-D depression**	
	**Unadjusted model**	**Fully-adjusted model**	**Unadjusted model**	**Fully-adjusted model**
Care type (ref: solo-care)				
Shared informal	-0.169 (0.156)	-0.110 (0.145)	-0.176 (0.179)	-0.131 (0.169)
Shared formal	0.002 (0.150)	0.191 (0.150)	-0.230 (0.202)	-0.034 (0.192)
Outsourced informal	0.362 (0.123) **	0.311 (0.117) **	0.259 (0.159) ~	0.061 (0.138)
Outsourced formal	-0.002 (0.200)	0.067 (0.188)	-0.098 (0.237)	0.046 (0.206)
Female (ref: male)	-0.081 (0.082)	0.004 (0.079)	-0.822 (0.105) ***	-0.780 (0.096) ***
Care type*gender				
Shared informal*female	0.198 (0.209)	0.267 (0.198)	-0.002 (0.250)	0.023 (0.234)
Shared formal*female	-0.362 (0.190) ~	-0.367 (0.184) *	-0.327 (0.260)	-0.335 (0.246)
Outs. informal*female	-0.125 (0.191)	-0.161 (0.175)	-0.032 (0.266)	0.036 (0.224)
Outs. formal*female	0.134 (0.248)	0.158 (0.232)	0.139 (0.354)	0.290 (0.306)
**Individual controls**				
Age		0.022 (0.004) ***		0.005 (0.005)
Education (ref: low)				
Intermediate		-0.022 (0.074)		0.078 (0.095)
High		-0.077 (0.092)		-0.025 (0.117)
Working (ref: no)		0.338 (0.099) ***		0.242 (0.127) ~
1+ ADL lim. (ref: no)		-0.214 (0.095) *		-0.619 (0.118) ***
1+ IADL lim (ref: no)		-0.256 (0.082) **		-0.857 (0.104) ***
Poor S.R. health (ref: no)		-1.041 (0.106) ***		-1.649 (0.122) ***
Social activities (ref: none)				
1		0.308 (0.073) ***		0.160 (0.090) ~
2 or more		0.431 (0.082) ***		0.115 (0.112)
**Partner controls**				
n. ADL		0.007 (0.026)		-0.069 (0.032) *
n. IADL		-0.098 (0.024) ***		-0.036 (0.028)
Poor S.R. health (ref: no)		-0.284 (0.073) ***		-0.200 (0.092) *
n. diagnosed conditions		-0.000 (0.020)		-0.052 (0.024) *
Low cognitive fcn. (ref: no)		0.122 (0.113)		0.039 (0.128)
**Couple controls**				
Log financial wealth		0.012 (0.006) *		0.021 (0.007) **
Log income		0.111 (0.041) **		0.069 (0.055)
Home ownership (ref: no)		0.236 (0.081) **		0.227 (0.093) *
Parent status (ref: childless)				
All children outside hh		0.233 (0.138) ~		0.292 (0.168) ~
Co-resident child(ren)		0.142 (0.148)		0.323 (0.182) ~
**Control for country of residence**	yes	yes	yes	yes
**Number of observations**	3,465	3,465	3,465	3,465

***, **, *, and ~ indicate p < 0.001, p < 0.01, p < 0.05, and p < 0.10, respectively. Source: SHARE wave 6 (2015).

Supplementary table 6. Figure 2 (life satisfaction): corresponding regression coefficients with robust standard errors in parentheses

**Life satisfaction**				
	**North**	**West**	**South**	**East**
Care type (ref: solo-care)				
Shared informal	-0.641 (0.345) ~	-0.061 (0.322)	-0.184 (0.250)	-0.141 (0.231)
Shared formal	-0.831 (0.490) ~	0.530 (0.202) **	0.172 (0.316)	-0.041 (0.338)
Outsourced informal	0.508 (0.304) ~	0.380 (0.199) ~	0.475 (0.210) *	0.036 (0.237)
Outsourced formal	-0.566 (0.647)	0.412 (0.217) ~	-0.214 (0.418)	0.055 (0.511)
Female (ref: male)	-0.076 (0.226)	-0.029 (0.118)	0.027 (0.171)	-0.011 (0.147)
Care type*gender				
Shared informal*female	1.456 (0.796) ~	-0.298 (0.433)	0.590 (0.345) ~	0.295 (0.312)
Shared formal*female	-0.327 (0.630)	-0.491 (0.248) *	-0.734 (0.400) ~	0.148 (0.420)
Outs. informal*female	0.633 (0.494)	0.117 (0.314)	-0.845 (0.335) *	0.272 (0.311)
Outs. formal*female	1.058 (0.766)	-0.122 (0.289)	0.402 (0.478)	-0.126 (0.740)
**Individual controls**				
Age	0.014 (0.015)	0.018 (0.007) **	0.023 (0.008) **	0.024 (0.008) **
Education (ref: low)				
Intermediate	-0.099 (0.252)	-0.214 (0.111) ~	-0.157 (0.187)	0.130 (0.129)
High	-0.484 (0.303)	-0.292 (0.128) *	0.160 (0.235)	0.158 (0.185)
Working (ref: no)	0.123 (0.278)	0.286 (0.149) ~	0.294 (0.224)	0.526 (0.196) **
1+ ADL lim. (ref: no)	0.155 (0.458)	-0.217 (0.146)	-0.266 (0.186)	-0.171 (0.181)
1+ IADL lim (ref: no)	-0.661 (0.354) ~	-0.112 (0.133)	-0.308 (0.156) *	-0.279 (0.154) ~
Poor S.R. health (ref: no)	-0.964 (0.595)	-1.084 (0.211) ***	-1.195 (0.185) ***	-0.846 (0.180) ***
Social activities (ref: none)				
1	-0.086 (0.261)	0.192 (0.113) ~	0.510 (0.145) ***	0.317 (0.145) *
2 or more	0.073 (0.244)	0.518 (0.110) ***	0.227 (0.249)	0.495 (0.193) *
**Partner controls**				
n. ADL	0.084 (0.122)	-0.031 (0.039)	-0.034 (0.051)	0.062 (0.047)
n. IADL	-0.011 (0.072)	-0.077 (0.037) *	-0.117 (0.043) **	-0.112 (0.043) **
Poor S.R. health (ref: no)	-0.469 (0.258) ~	-0.271 (0.124) *	-0.111 (0.139)	-0.370 (0.136) **
n. diagnosed conditions	0.003 (0.088)	0.014 (0.029)	0.001 (0.139)	-0.001 (0.036)
Low cognitive fcn. (ref: no)	-0.313 (0.312)	0.342 (0.188) ~	0.335 (0.190) ~	-0.081 (0.226)
**Couple controls**				
Log financial wealth	0.009 (0.015)	0.021 (0.010) *	0.017 (0.012)	-0.000 (0.011)
Log income	0.179 (0.246)	0.153 (0.073) *	0.154 (0.060) *	0.040 (0.093)
Home ownership (ref: no)	0.062 (0.242)	0.094 (0.107)	0.704 (0.228) **	0.168 (0.155)
Parent status (ref: childless)				
All children outside hh	0.286 (0.411)	0.030 (0.204)	0.067 (0.240)	0.610 (0.331) ~
Co-resident child(ren)	0.194 (0.479)	-0.084 (0.226)	0.141 (0.252)	0.415 (0.346)
**Control for country of residence**	yes	yes	yes	yes
**Number of observations**	247	1,056	990	1,172

***, **, *, and ~ indicate $p < 0.001$, $p < 0.01$, $p < 0.05$, and $p < 0.10$, respectively. Source: SHARE wave 6 (2015).

Supplementary table 7. Figure 3 (reverse-coded EURO-D): corresponding regression coefficients with robust standard errors in parentheses

**Reverse-coded EURO-D**				
	**North**	**West**	**South**	**East**
Care type (ref: solo-care)				
Shared informal	-0.808 (0.734)	-0.189 (0.282)	-0.048 (0.380)	-0.173 (0.240)
Shared formal	-0.723 (0.448) ~	0.062 (0.265)	-0.220 (0.448)	0.231 (0.400)
Outsourced informal	0.521 (0.290) ~	0.081 (0.263)	0.117 (0.276)	0.033 (0.228)
Outsourced formal	-0.241 (0.604)	-0.289 (0.272)	0.349 (0.498)	0.679 (0.377) ~
Female (ref: male)	-0.478 (0.283) ~	-0.878 (0.165) ***	-0.596 (0.212) **	-1.008 (0.163) ***
Care type*gender				
Shared informal*female	0.598 (1.112)	0.058 (0.457)	-0.020 (0.505)	0.195 (0.329)
Shared formal*female	-1.213 (0.632) *	0.065 (0.343)	-0.927 (0.575) ~	-0.354 (0.522)
Outs. informal*female	-0.067 (0.620)	0.569 (0.355)	-0.527 (0.462)	0.304 (0.370)
Outs. formal*female	0.629 (0.731)	0.224 (0.436)	0.486 (0.664)	-0.844 (0.685)
**Individual controls**				
Age	0.003 (0.017)	0.020 (0.008) *	0.003 (0.011)	- 0.002 (0.009)
Education (ref: low)				
Intermediate	0.110 (0.272)	-0.018 (0.159)	0.220 (0.259)	0.091 (0.151)
High	0.034 (0.312)	-0.353 (0.189) ~	0.056 (0.302)	0.281 (0.209)
Working (ref: no)	-0.232 (0.390)	0.355 (0.197) ~	0.031 (0.322)	0.477 (0.221) *
1+ ADL lim. (ref: no)	0.468 (0.447)	-0.161 (0.180)	-1.118 (0.232) ***	-0.746 (0.213) ***
1+ IADL lim (ref: no)	-1.560 (0.422) ***	-0.867 (0.170) ***	-1.004 (0.216) ***	-0.649 (0.177) ***
Poor S.R. health (ref: no)	-1.285 (0.427) **	-1.926 (0.262) ***	-1.647 (0.235) ***	-1.464 (0.192) ***
Social activities (ref: none)				
1	0.069 (0.281)	0.164 (0.146)	0.077 (0.196)	0.281 (0.157) ~
2 or more	0.264 (0.286)	0.319 (0.157) *	-0.557 (0.372)	0.277 (0.219)
**Partner controls**				
n. ADL	0.184 (0.109) ~	-0.065 (0.060)	-0.062 (0.066)	-0.085 (0.050) ~
n. IADL	-0.067 (0.082)	-0.038 (0.052)	-0.070 (0.056)	-0.013 (0.044)
Poor S.R. health (ref: no)	0.019 (0.313)	-0.405 (0.164) *	-0.319 (0.193) ~	-0.020 (0.151)
n. diagnosed conditions	-0.192 (0.091) *	-0.032 (0.041)	-0.085 (0.053)	-0.017 (0.039)
Low cognitive fcn. (ref: no)	-0.474 (0.386)	-0.142 (0.237)	0.119 (0.244)	0.259 (0.205)
**Couple controls**				
Log financial wealth	0.014 (0.018)	0.027 (0.013) *	0.035 (0.016) *	0.002 (0.012)
Log income	0.064 (0.312)	-0.067 (0.100)	0.174 (0.083) *	0.046 (0.104)
Home ownership (ref: no)	0.354 (0.250)	0.256 (0.140) ~	0.209 (0.255)	0.226 (0.165)
Parent status (ref: childless)				
All children outside hh	0.452 (0.493)	0.096 (0.241)	0.035 (0.326)	0.738 (0.348) *
Co-resident child(ren)	0.847 (0.553)	0.120 (0.274)	0.198 (0.342)	0.651 (0.369) ~
**Control for country of residence**	yes	yes	yes	yes
**Number of observations**	247	1,056	990	1,172

***, **, *, and ~ indicate p < 0.001, p < 0.01, p < 0.05, and p < 0.10, respectively. Source: SHARE wave 6 (2015).

Supplementary figure 1: Predicted scores for life satisfaction by I's gender and care context (fully-adjusted models). All covariates held at observed values. Analyses restricted to couples where partners' reports of care are concordant (n = 2,831)

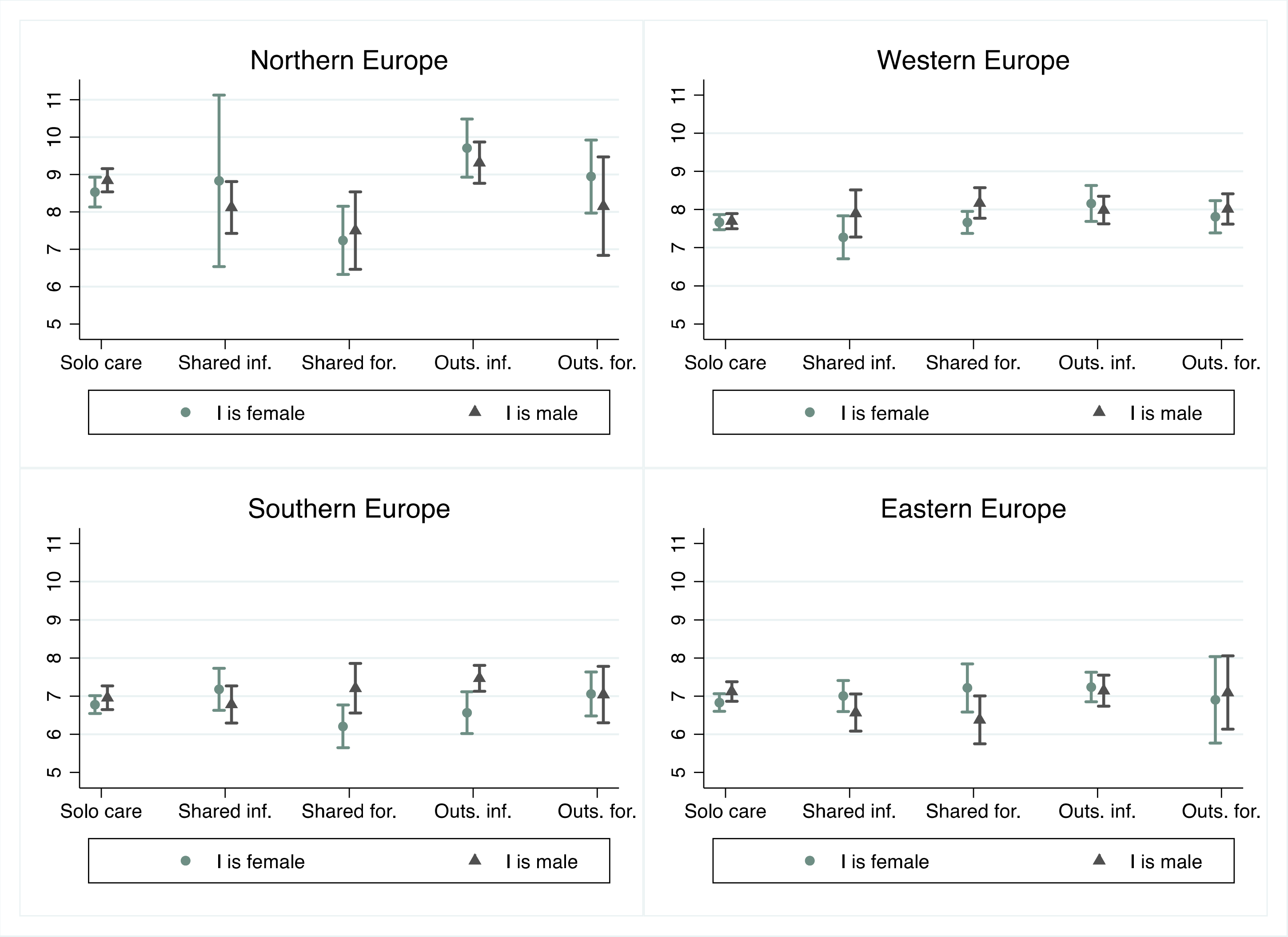

Source: SHARE wave 6 (2015).

Supplementary figure 2. Predicted scores for reverse EURO-D by I's gender and care context (fully-adjusted models). All covariates held at observed values. Analyses restricted to couples where partners' reports of care are concordant (n = 2,831).

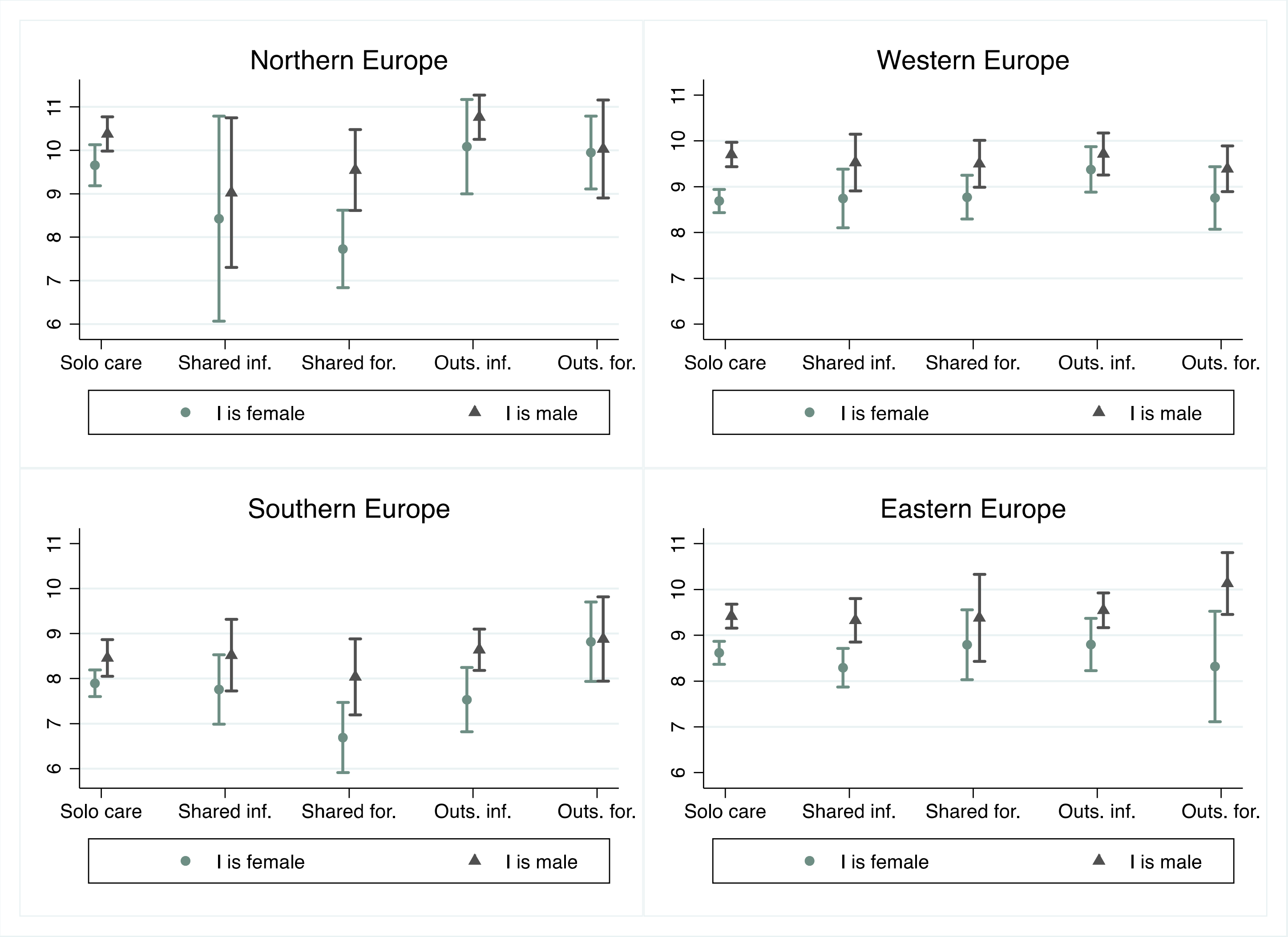

Source: SHARE wave 6 (2015).
